# Epidemiological and geographical distribution profile of urban sporotrichosis in the city of São Paulo^☆^

**DOI:** 10.1016/j.abd.2020.11.014

**Published:** 2022-01-05

**Authors:** John Verrinder Veasey, Gustavo de Sá Menezes Carvalho, Ligia Rangel Barboza Ruiz, Milton Ferreira Neves Neto, Clarisse Zaitz

**Affiliations:** a Dermatology Clinic, Hospital da Santa Casa de São Paulo, São Paulo, SP, Brazil; b Faculdade de Ciências Médicas, Santa Casa de São Paulo, São Paulo, SP, Brazil

**Keywords:** Epidemiology of infectious diseases, Health and environment, Healthcare policies, Sporotrichosis

## Abstract

**Background:**

Sporotrichosis is the most frequent subcutaneous mycosis in Latin America, where it is considered endemic. At the end of the 20th century, the first cases of zoonotic transmission were described in Rio de Janeiro, triggering an epidemic outbreak that spread to other regions of Brazil. The lack of disease notification omits its real occurrence in the country, which happens in its most populous city, São Paulo.

**Objective:**

To evaluate the epidemiological aspects of the patients seen at a hospital in São Paulo aiming at establishing the geographic distribution of this disease.

**Methods:**

This is a retrospective study that analyzed data from medical records of patients with a clinical and laboratory diagnosis of sporotrichosis attended at a tertiary hospital in the city of São Paulo between 2012 and 2020.

**Results:**

Twenty patients were included. As for zoonotic surveillance, 30% denied contact with an animal, and 70% reported previous contact with a sick cat, with no other animals being mentioned. One case was allochthonous and the others autochthonous, showing a dissemination behavior from a focus in the eastern area of the capital.

**Study limitations:**

The present study was based on data from only one hospital. Studies that include data from other hospitals and other regions must be carried out to obtain a complete picture of this disease.

**Conclusions:**

As in other regions of the country, zoonotic sporotrichosis presents itself as an endemic disease with an increase in the number of cases. The findings highlight epidemiological characteristics of great importance so that public health policies can contain disease progression.

Sporotrichosis is a subacute or chronic infection caused by dimorphic fungi of the genus *Sporothrix*.^1^ The classic form occurs when the fungus is inoculated into the skin or mucosa from contaminated plant material. However, from the 1990s onwards, feline zoonotic transmission became endemic in Brazil.^1^ Outbreaks have been reported in several regions of the country,^2^, ^3^, ^4^ but little is known about the behavior of this mycosis in its most populous city, São Paulo, where it is not a disease of mandatory notification.

This study presents epidemiological data of cases attended at a hospital in downtown São Paulo between 2012 and 2020, aiming to elucidate disease distribution. All cases with clinical suspicion of sporotrichosis that were confirmed through the gold standard diagnostic method, a positive culture for fungi from lesion secretions or fragments, were included in the study. For each included case, the following clinical and epidemiological characteristics were analyzed: age, sex, origin, previous contact with a sick animal, and year of diagnosis. The study was submitted to the institution’s Research Ethics Committee and approved according to CAAE n. 35820920.4.0000.5479.

Twenty patients were included, aged two to 81 years (mean 32.2 ± 25.10 years), 55% females and 45% males. As for zoonotic surveillance, 30% denied contact with an animal, and 70% reported the previous contact with a sick cat, with no other animals being mentioned.

As for the epidemiological aspects, there were 19 autochthonous cases from greater São Paulo and one allochthonous case (from the state of Bahia). There was a progressive increase in the number of autochthonous cases over the years: one in 2012, none in 2013, one in 2014, two per year in 2015 to 2017, three in 2018, five in 2019, and three in the first four months of 2020. Over the years, there was a progression from the initial cases located in neighborhoods in the eastern area of São Paulo to neighborhoods in other areas of the city, as well as to the neighboring municipalities that constitute Greater São Paulo, such as Poá, Guarulhos, Ferraz de Vasconcelos and Diadema (Fig. 1).

**Figure 1:**
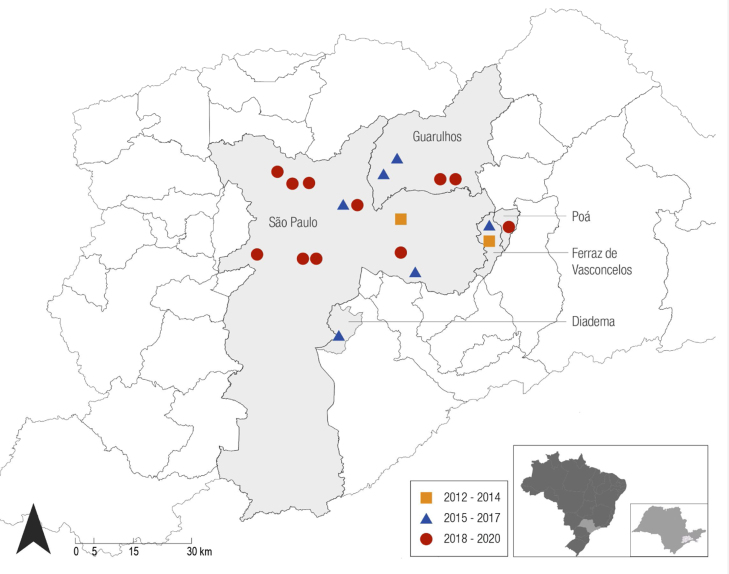
Distribution of autochthonous cases in Greater São Paulo. The locations identified on the map follow the progression in the three-year periods: two cases from 2012 to 2014, six cases from 2015 to 2017, and eleven cases from 2018 to 2020.

When analyzing the described epidemiological aspects, it is noteworthy that most patients (70%) reported contact with sick cats. Another fact to be highlighted is that the majority of cases (60%) occurred in patients at the extremes of age, corresponding to the population with greater contact with sick animals, compatible with what is described in the literature.^1^

The zoonotic transmission of sporotrichosis by contaminated animals, especially felines, has been a matter of concern in several regions of the country. In the 1990s, the first publications on the epidemic appeared in the state of Rio de Janeiro, followed by a considerable increase in the number of cases in felines and humans over the years, making it currently be considered hyperendemic in the state.^1^ Since 2013, notification has become mandatory in the state of Rio de Janeiro, which does not happen in other states in Brazil; therefore, epidemiological estimates are obtained mainly based on cases reported in the literature, underestimating the actual situation found in these places. Recently, other states in the southern and southeastern regions of Brazil, such as Rio Grande do Sul^3^ and Espírito Santo,^4^ reported an epidemic situation of sporotrichosis, indicating disease progression throughout the country, and even affecting neighboring countries, such as Argentina.^2^

The emergence and spread of sporotrichosis cases in Brazil have been neglected for several years, making it a frequent and uncontrolled disease that was previously rare in many regions. Ongoing socio-economic and environmental difficulties, with urban agglomeration and poor basic sanitation, together with scarce and inadequate health services, are promoting this expansion.

In São Paulo, there have been few studies that mapped the disease progression in humans. In 2011, an outbreak of zoonotic sporotrichosis was detected in the administrative district of Itaquera, in the eastern area of São Paulo, with a number of feline cases of sporotrichosis known to be emerging in this region.^5^ The geographic progression of cases in humans diagnosed from that date onwards suggests a possible spread from the focus in the eastern area of the city to other neighborhoods and neighboring cities.

Public health actions aimed at improving human and animal health are required, including educational programs for health professionals that will help to increase the clinical suspicion of human and feline disease, so that infected animals can be screened. More reliable epidemiological data on the prevalence and incidence can be obtained through mandatory reporting of cases, thus facilitating the work of the epidemiological surveillance by zoonoses center to eradicate the disease and control new cases.^3^, ^4^ Moreover, actions to promote the availability of easily accessible veterinary services and antifungal treatment for the population of pets are necessary, as well as better control programs for stray animals and the correct destiny of animals that die due to sporotrichosis, since the fungus is geophilic and ends up perpetuating itself in the environment when the dead animal is dumped in vacant lots or the animal is buried.^3^, ^4^, ^5^

Socio-economic and environmental difficulties, associated with the lack of policies such as the population awareness and free access to examination and treatment of animals, have contributed to the spread of the disease. This study highlights epidemiological characteristics of great importance, reaffirming previous concepts and presenting ideas that should be analyzed in future similar studies in the city, aiming to obtain greater knowledge and control of the disease.

## Financial support

None declared.

## Authors’contributions

John Verrinder Veasey: Design and planning of the study; drafting and editing of the manuscript; collection, analysis, and interpretation of data; effective participation in research orientation; intellectual participation in the propaedeutic and/or therapeutic conduct of the studied cases; critical review of the literature; critical review of the manuscript; approval of the final version of the manuscript.

Gustavo de Sá Menezes Carvalho: Collection, analysis, and interpretation of data; approval of the final version of the manuscript.

Ligia Rangel Barbosa Ruiz: Intellectual participation in the propaedeutic and/or therapeutic conduct of the studied cases; critical review of the literature; critical review of the manuscript; approval of the final version of the manuscript.

Milton Ferreira Neves Neto: Drafting and editing of the manuscript; collection, analysis, and interpretation of data.

Clarisse Zaitz: Intellectual participation in the propaedeutic and/or therapeutic conduct of the studied cases; critical review of the literature; critical review of the manuscript; approval of the final version of the manuscript.

## Conflicts of interest

None declared.
